# Structure Enhancement Relationship of Chemical Penetration Enhancers in Drug Transport across the Stratum Corneum

**DOI:** 10.3390/pharmaceutics4010071

**Published:** 2012-01-17

**Authors:** Doungdaw Chantasart, S. Kevin Li

**Affiliations:** 1 Department of Pharmacy, Faculty of Pharmacy, Mahidol University, Bangkok 10400, Thailand; 2 Division of Pharmaceutical Sciences, College of Pharmacy, University of Cincinnati, Cincinnati, OH 45267, USA

**Keywords:** skin penetration enhancers, transdermal drug delivery, n-octanol-water partition coefficient, penetration enhancement, skin

## Abstract

The stratum corneum is a major barrier of drug penetration across the skin in transdermal delivery. For effective transdermal drug delivery, skin penetration enhancers are used to overcome this barrier. In the past decades, a number of research studies were conducted to understand the mechanisms of skin penetration enhancers and to develop a structure enhancement relationship. Such understanding allows effective prediction of the effects of skin penetration enhancers, assists topical and transdermal formulation development, and avoids extensive enhancer screening in the transdermal delivery industry. In the past two decades, several hypotheses on chemical enhancer-induced penetration enhancement for transport across the skin lipoidal pathway have been examined based on a systematic approach. Particularly, a hypothesis that skin penetration enhancement is directly related to the concentration of the enhancers in the stratum corneum lipid domain was examined. A direct relationship between skin penetration enhancer potency (based on enhancer aqueous concentration in the diffusion cell chamber) and enhancer n-octanol-water partition coefficient was also established. The nature of the microenvironment of the enhancer site of action in the stratum corneum lipid domain was found to be mimicked by n-octanol. The present paper reviews the work related to these hypotheses and the relationships between skin penetration enhancement and enhancer concentration in the drug delivery media and stratum corneum lipids.

## 1. Introduction

Transdermal drug delivery offers a number of advantages compared with the conventional routes of drug administration. However, the barrier function of the skin outermost layer, stratum corneum (SC) is the major limitation to this utility [1]. Overcoming this barrier by using skin penetration enhancers has been one of the great interests in pharmaceutical research. Skin penetration enhancers are defined as chemicals which are themselves pharmaceutically inert, but can partition into and interact with the barrier of the stratum corneum when combined in a transdermal formulation, thereby reducing the skin resistance to drug transport [2,3,4]. Accordingly, the mechanism of action of skin penetration enhancers has become a topic of interest for many researchers. During the past several decades, there have been a large number of research reports [5,6,7,8,9,10,11,12], review articles [4,13,14,15], patents [16,17] and books [18,19] published on the topics. These studies have improved our knowledge in the field of penetration enhancers. Over the past two decades, a research focus of Higuchi’s group and later in our group was the study of the mechanism of action of skin penetration enhancers to gain better insights into the relationship between the nature of the enhancer molecule and the enhancer effectiveness [6,20,21,22,23,24,25,26,27]. Such research work was previously summarized in two reviews in 2006 [28,29]. Since the publication of these review chapters, a number of advances in skin penetration enhancer research have been made. They include those from our studies (e.g., [30,31]) and studies from other groups [32,33,34,35] on the structure-enhancement activity relationship of penetration enhancers in transdermal transport. A complete review on penetration enhancers will not be provided here due to the extensive literature on this topic. The present review paper will focus on summarizing the findings, hypotheses, and conclusions of the research studies in the past five years from our group built on those of similar studies in the fifteen years from 1990 to 2005. 

This paper is separated into three main sections. The paper first describes the physical model and experimental approach in skin permeability studies used in our laboratories. Subsequently, the relationship between skin penetration enhancer potencies and enhancer physicochemical properties (*i.e.*, n-octanol-water partition coefficient) will be discussed. The relationship between flux enhancement effect of skin penetration enhancers and their concentration in stratum corneum lipids will be reviewed. We will also discuss the nature of the microenvironment at the enhancer site of action and how it influences the effectiveness of skin penetration enhancers. Lastly, the possible mechanism of action of skin penetration enhancers is provided.

## 2. Physical Model and Experimental Approaches in Chemical Enhancer Studies

### 2.1. Parallel Pathway and Equilibrium Approach

An approach to study the mechanisms of skin penetration enhancers is the skin parallel pore and lipoidal pathway transport model. In the past two decades, we have used this model approach to examine the effects of skin penetration enhancers and to study the structure enhancement relationship of skin penetration enhancers in transdermal transport [6,22,23,24,25,26,27,30,31]. In this parallel pathway transport model, the permeability coefficient of the stratum corneum (*P_SC_*) is divided into two components:

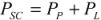
(1)
where *P_P_* and *P_L_* are the permeability coefficients for the pore pathway and the lipoidal pathway in the stratum corneum, respectively. The permeability of the pore pathway and its contribution to permeant transport across the stratum corneum can be estimated using a polar probe permeant such as tetraethylammonium (TEA). The lipoidal transport pathway across stratum corneum is generally viewed as the intercellular lipid domain in the stratum corneum. The relationship between the permeability coefficient of the stratum corneum and the total skin permeability coefficient (full-thickness or split-thickness skin) can be expressed as:

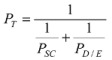
(2)
where *P_T_* is the total skin permeability coefficient and *P_D/E_* is the permeability coefficient of the viable epidermis and dermis combination. The permeability coefficient of the viable epidermis and dermis (*P_D/E_*) can be determined from experiments of tape-stripped skin. Substituting Equation (1) into Equation (2) yields:

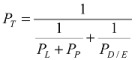
(3)
For moderately lipophilic permeants with octanol/phosphate buffered saline (PBS) partition coefficients (*K*_octanol/PBS_) in the order of 10^2^, *P_L_* becomes the determining factor for *P_T_* in Equation (3). Particularly, based on the results from previous studies [22,23], the use of corticosterone (log *K*_octanol/PBS_ ~ 2.0) as the probe permeant allows Equation (3) to be approximated by:

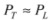
(4)
Note that octanol/PBS partition coefficient and octanol/water partition coefficient are used interchangeably in this review paper as these two quantities are essentially the same for uncharged compounds. For other steroidal permeants, *P_L_* can be calculated using Equation (3) with *P_D/E_* and *P_P_* values determined separately in transport experiments with stripped skin and TEA, respectively. Model analyses that separate the effects of chemical enhancers on permeant transport across the pore and lipoidal transport pathways allow the mechanistic studies of enhancement effects of skin penetration enhancers. This approach is essential in determining the relationship between the physicochemical properties of the enhancers and enhancer effectiveness and for predicting the effects of penetration enhancers in transdermal drug delivery. The effects of skin penetration enhancers on transport across the lipoidal pathway of the stratum corneum will be the focus of this review article. 

### 2.2. Symmetric and Asymmetric Conditions

*In vitro* penetration enhancer studies in the literature commonly employ an “asymmetric” approach (asymmetric condition) where the skin transport experiments are conducted with the enhancer solution and the drug applied to the donor chamber and buffered saline in the receiver chamber of the diffusion cells [20,21,23]. This asymmetric condition generally leads to an enhancer concentration gradient (or activity) across the stratum corneum; a situation in which the local penetration enhancement induced by the enhancer varies with the position within the stratum corneum. To provide direct assessment of the effectiveness of penetration enhancers and establish a quantitative structure enhancement relationship for skin transport, a symmetric and equilibrium configuration was employed in our studies (symmetric condition). In this symmetric configuration, the enhancer is present at equal concentrations in both the donor and receiver chambers of a side-by-side diffusion cell and in equilibrium with skin [23,24,25,26,27,30,31]. The complications arising from enhancer concentration gradients across the membrane [20,21] can therefore be avoided under this condition. The permeability coefficients obtained for the permeants with an enhancer can be used directly to determine the effectiveness of the enhancer (enhancer potency) in skin penetration enhancement, understand the mechanisms of the enhancers, and establish a structure-enhancement relationship. However, the main disadvantage of this approach is that the symmetric condition is different from the asymmetric condition normally encountered in practice; it does not mimic transdermal delivery directly. 

**Figure 1:**
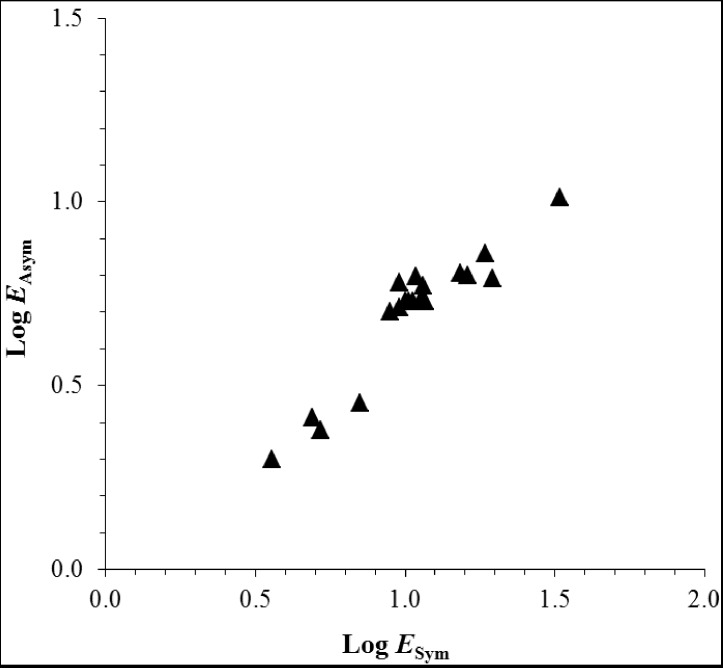
Relationship between the enhancement factors under the asymmetric conditions (*E*_Asym_) and those under the symmetric conditions (*E*_Sym_). Replotted using the data from Chantasart and Li [36].

A recent study has examined the differences between chemical permeation experiments under the asymmetric and symmetric conditions [36]. In this study, the effects of penetration enhancers, 1-butanol, 1-pentanol, 1-hexanol, 1-octanol, 2-phenylethanol, thymol, carvacrol, 1-hexyl-2-pyrrolidone, 1-octyl-2-pyrrolidone, and 1-octyl-2-azacycloheptanone upon the transport of corticosterone across human epidermal membrane (HEM) under the asymmetric and symmetric conditions were compared. Figure 1 is a plot of the experimental enhancement factors under the asymmetric conditions against those under the symmetric conditions in this study. The results show a correlation between transdermal penetration enhancement under the asymmetric and symmetric conditions. This correlation implies that the findings in the symmetric transport studies can be extrapolated to the asymmetric condition and supports the practical usefulness of the symmetric experimental approach. The mechanisms of the enhancers for skin penetration under the asymmetric and symmetric conditions are also likely to be the same. 

### 2.3. Solvent Systems in Penetration Enhancer Studies

Skin penetration enhancer studies commonly employ cosolvents and/or solubilizing agents because most chemical enhancers present in transdermal products are highly lipophilic with low aqueous solubility. Examples of cosolvents are ethanol and propylene glycol. A limitation of these enhancer studies to investigate the mechanisms of penetration enhancers and to establish a general quantitative structure enhancement relationship is the potential of cosolvents to alter the barrier properties of the stratum corneum. In addition, it is difficult to delineate potential synergistic effects between the enhancers and cosolvents for mechanistic interpretation of the enhancer effects [37,38]. As a result, penetration enhancer studies by Higuchi *et al*. in the 1990s used buffered saline without cosolvents to avoid possible synergy effects from cosolvents in order to examine the sole effects of the enhancers under the symmetric condition [6,22]. Later, similar studies have successfully used this aqueous medium approach to study the mechanisms of penetration enhancers [23,24,25,26,27,30,31]. 

A disadvantage of the aqueous medium approach (without cosolvents) is the inability of the system to dissolve highly lipophilic enhancers in the experiments and the rapid depletion of the enhancers in the aqueous medium when the skin is equilibrated with the enhancer solutions [39]. To avoid this problem, a series of transdermal transport studies was conducted using the “maximum intrinsic enhancement” approach to evaluate the effectiveness of highly lipophilic skin penetration enhancers with, e.g., *K*_octanol/PBS_ > 3 [40]. This approach allows the comparison of enhancer effectiveness when the enhancers are presented at their highest thermodynamic activity in equilibrium with the stratum corneum. Briefly, these studies involve the direct immersion of the skin in the liquid enhancers; this procedure provided an infinite dose of the enhancer and allowed the equilibrium of the enhancer in its pure state with the skin [40,41]. After equilibration, the skin membrane was mounted in side-by-side diffusion cells for the permeation experiments with aqueous donor and receiver solution (*i.e.*, PBS) to assess the effectiveness of the enhancers. A moderately lipophilic permeant corticosterone was used to probe the lipoidal pathway in the stratum corneum. Because of the lipophilicity of the enhancers, depletion of the enhancers from the stratum corneum to the aqueous solution in the diffusion cell chambers was expected to be minimal and constant enhancer concentration can therefore be maintained in the stratum corneum. The results of these maximum enhancement studies will be discussed later in this paper. The disadvantage of this approach is the lack of control of enhancer concentration in the diffusion cell chamber (*i.e.*, always at saturation in the aqueous solution) and hence in the skin, so this approach cannot evaluate the effects of enhancer concentration upon skin penetration enhancement. 

### 2.4. Comparison of the Effects of Skin Penetration Enhancer on Human Epidermal Membrane (HEM) and Hairless Mouse Skin (HMS)

**Figure 2:**
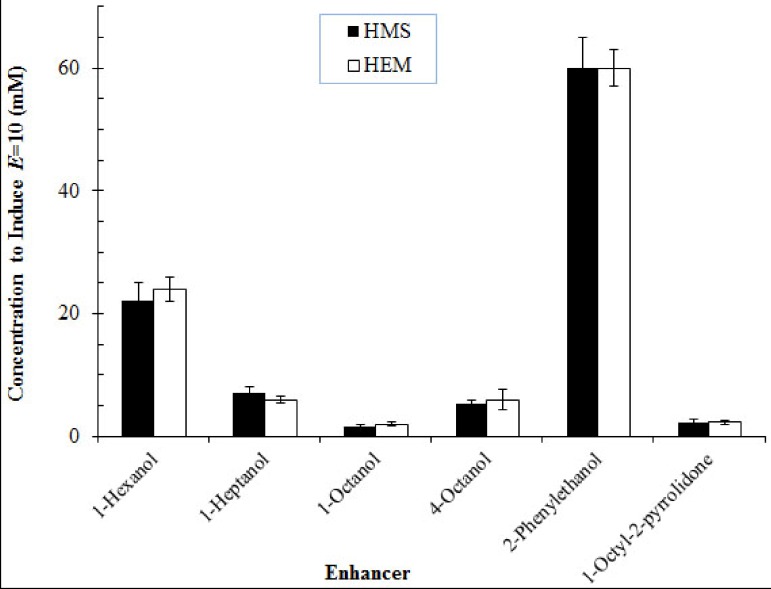
Representative results in the hairless mouse skin (HMS) and human epidermal membrane (HEM) studies. Isoenhancement concentrations (mM) of *E* = 10 obtained with HMS and HEM for the enhancers in phosphate buffered saline (PBS). Isoenhancement concentrations are defined as the aqueous concentrations for which different enhancers induce the same extent of penetration enhancement, *E*, for transport across the lipoidal pathway of stratum corneum. The data are taken from Chantasart *et al*. [30].

**Figure 3:**
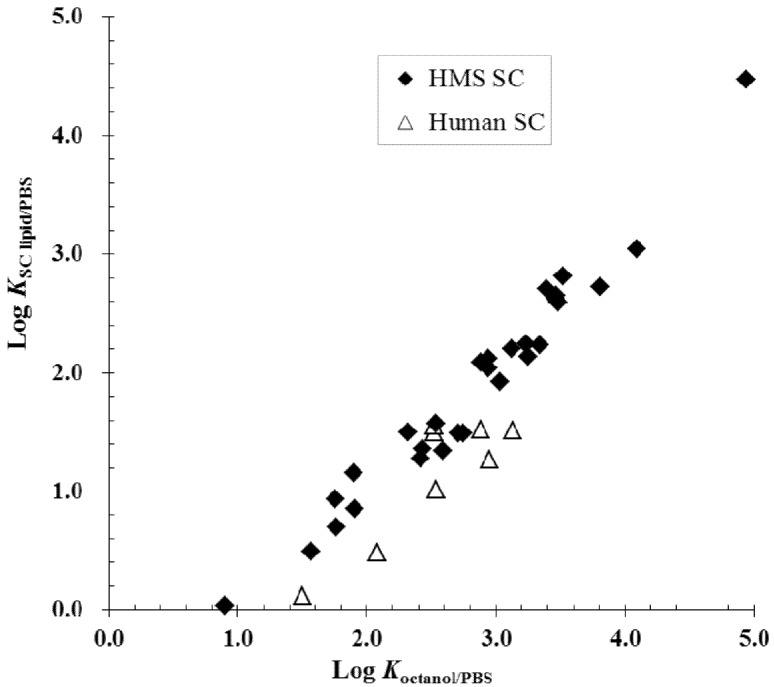
Relationship between *K*_ SC lipid/PBS_ (the intercellular lipid/PBS partition coefficients) of the enhancers and *K*_octanol/PBS_ (the enhancer n-octanol/PBS partition coefficients) in hairless mouse skin stratum corneum (HMS SC) and human stratum corneum (human SC). The data are taken from previous hairless mouse skin (HMS) and human epidermal membrane (HEM) studies [6,22,23,24,25,26,27,30,31]. PBS = phosphate buffered saline.

Skin penetration enhancer studies on the lipoidal pathway of stratum corneum and quantitative structure enhancement relationship using the equilibrium and symmetric approach have been conducted using both hairless mouse skin and human skin. Due to the well-known differences between mouse and human skin, the effects of enhancers on human epidermal membrane were compared with those of hairless mouse skin under the same experimental setup and the symmetric conditions [30]. In this study, the effects of enhancers on the permeability coefficients of the lipoidal pathways of human skin for corticosterone and their respective penetration enhancement factors were found to be essentially the same as those of hairless mouse skin (Figure 2). These results suggest that for the assessment of skin permeation utilizing the lipoidal pathway, hairless mouse skin can be a reliable model for human skin. A correlation between the intercellular lipid/PBS partition coefficients of the enhancers (*K*_SC__lipid/PBS_) and their *K*_octanol/PBS_ was also observed in the human skin study, which is consistent with those observed in the previous hairless mouse skin studies (Figure 3). This suggests that hairless mouse skin is useful in the mechanistic studies of the effects of skin penetration enhancers on the lipoidal pathway of human stratum corneum. The following sections summarize the findings to date in our development of a structure enhancement relationship of skin penetration enhancers using the aforementioned experimental approaches.

## 3. Structure Activity Relationship of Skin Penetration Enhancers

Table 1 lists the chemical enhancers studied in our groups and in collaboration with other groups to investigate the mechanisms of penetration enhancers and to examine a possible quantitative structure enhancement relationship for drug penetration across the stratum corneum lipoidal pathway. In these studies, the relationships between enhancer potency and *K*_octanol/PBS_ based on enhancer concentration in aqueous solutions, enhancer concentration in the stratum corneum lipids, and enhancer maximum enhancement effect when the enhancers are in equilibrium with the stratum corneum were investigated. The microenvironment of the site of action of skin penetration enhancers in the stratum corneum lipoidal pathway for transdermal permeation was also studied. This review paper provides a general overview of these studies.

**Table 1:** Skin penetration enhancers studied (listed alphabetically according to chemical classes).

Chemical	Log *K*_octanol/PBS_ or Log *K*_octanol/water_	Class	References
Ethanol	−0.30 ^a^	Alcohol	Kim *et al*. [6]
1-Propanol	0.25 ^a^	Alcohol	Kim *et al*. [6]
1-Butanol	0.84 ^a^	Alcohol	Kim *et al*. [6], Chantasart and Li [36]
1-Pentanol	1.5 ^a^	Alcohol	Kim *et al*. [6], Chantasart and Li [36]
1-Hexanol	2.1 ^b^	Alcohol	Warner *et al*. [24], Ibrahim and Li [40,56]
1-Heptanol	2.5 ^b^	Alcohol	Warner *et al*. [24], Chantasart *et al*. [27]
1-Octanol	3.1 ^b^	Alcohol	Warner *et al*. [24], Ibrahim and Li [40,56]
1-Nonanol	3.5 ^b^	Alcohol	Chantasart *et al*. [27]
1-Undecanol	4.2 ^c^	Alcohol	Ibrahim and Li [40,56]
Oleyl alcohol	7.0 ^d^	Alcohol	Ibrahim and Li [40,56]
2-Hexanol	1.83 ^b^	Alcohol	Chantasart *et al*. [27]
3-Hexanol	1.81 ^b^	Alcohol	Chantasart *et al*. [27]
2-Heptanol	2.43 ^b^	Alcohol	Chantasart *et al*. [27]
3-Heptanol	2.42 ^b^	Alcohol	Chantasart *et al*. [27]
4-Heptanol	2.32 ^b^	Alcohol	Chantasart *et al*. [27]
2-Octanol	3.03 ^b^	Alcohol	Chantasart *et al*. [27]
3-Octanol	2.94 ^b^	Alcohol	Chantasart *et al*. [27]
4-Octanol	2.88 ^b^	Alcohol	Chantasart *et al*. [27]
2-Nonanol	3.48 ^b^	Alcohol	Chantasart *et al*. [27]
3-Nonanol	3.47 ^b^	Alcohol	Chantasart *et al*. [27]
4-Nonanol	3.46 ^b^	Alcohol	Chantasart *et al*. [27]
5-Nonanol	3.42 ^b^	Alcohol	Chantasart *et al*. [27]
cis-3-Penten-1-ol	1.08 ^e^	Alcohol	He *et al*. [26]
cis-3-Hexen-1-ol	1.63 ^e^	Alcohol	He *et al*. [26]
cis-3-Octen-1-ol	2.71 ^e^	Alcohol	He *et al*. [26]
cis-3-Nonen-1-ol	3.25 ^e^	Alcohol	He *et al*. [26]
trans-3-Hexen-1-ol	1.76 ^e^	Alcohol	He *et al*. [26]
trans-Hydroxyproline-*N*-decanamide-*C*-ethylamide	2.9 ^f^	Amide/Alcohol	Warner *et al*. [24]
*N*,*N*-Dimethylhexanamide	1.4 ^g^	Amide	Warner *et al*. [23]
*N*,*N*-Dimethylheptanamide	1.9 ^g^	Amide	Warner *et al*. [23]
*N*,*N*-Dimethyloctanamide	2.6 ^g^	Amide	Warner *et al*. [23]
*N*,*N*-Dimethylnonanamide	1.9 ^g^	Amide	Warner *et al*. [23]
Benzyl alcohol	1.1 ^d^	Aromatic ring: alcohol	Ibrahim and Li [40,56]
2-Phenoxyethanol	1.2 ^h^	Aromatic ring: alcohol	Ibrahim and Li [40,56]
2-Phenylethanol	1.6 ^i^	Aromatic ring: alcohol	Chantasart *et al*. [30]
Padimate O (PadO)	5.8 ^d^	Aromatic ring: aminobenzoate	Ibrahim and Li [40,56]
Butylated hydroxyanisole	3.1 ^j^	Aromatic ring: hydroxyanisole	Ibrahim and Li [40,56]
2-Ethylhexyl salicylate	6.0 ^d^	Aromatic ring: salicylate	Ibrahim and Li [40,56]
Salicylaldehyde	2.0 ^d^	Aromatic ring: salicylate	Ibrahim and Li [40,56]
1-Butyl-2-azacycloheptanones	1.8 ^f^	Azone	He *et al*. [25]
1-Hexyl-2-azacycloheptanones	2.9 ^f^	Azone	He *et al*. [25]
1-Octyl-2-azacycloheptanones	4.0 ^f^	Azone	He *et al*. [25]
Laurocapram (Azone)	6.3 ^k^	Azone	Ibrahim and Li [40,56]
1,2-Hexanediol	0.78 ^f^	Diol	Warner *et al*. [23]
1,2-Octanediol	2.1 ^f^	Diol	Warner *et al*. [23]
1-2-Decanediol	3.2 ^f^	Diol	Warner *et al*. [23]
2-(1-Butyl)-2-methyl-1,3 dioxolane	2.1 ^f^	Dioxolane	Warner *et al*. [24]
2-(1-Hexyl)-2-methyl-1,3 dioxolane	3.5 ^f^	Dioxolane	Warner *et al*. [24]
Isopropyl myristate	7.3 ^d^	Ester	Ibrahim and Li [40,56]
Decanoic acid	4.0 ^d^	Fatty acid	Ibrahim and Li [41]
Undecanoic acid	4.5 ^d^	Fatty acid	Ibrahim and Li [41]
Lauric acid	5.0 ^d^	Fatty acid	Ibrahim and Li [41]
Tridecanoic acid	5.5 ^d^	Fatty acid	Ibrahim and Li [41]
Myristic acid	6.0 ^d^	Fatty acid	Ibrahim and Li [41]
Pentadecanoic acid	6.5 ^d^	Fatty acid	Ibrahim and Li [41]
Palmitic acid	7.0 ^d^	Fatty acid	Ibrahim and Li [41]
Stearic acid	7.9 ^d^	Fatty acid	Ibrahim and Li [41]
Linoleic acid	7.5 ^d^	Fatty acid	Ibrahim and Li [41]
Oleic acid	7.6 ^d^	Fatty acid	Ibrahim and Li [40,41,56]
Ricinoleic acid	6.2 ^d^	Fatty acid	Ibrahim and Li [41]
1-Octyl-β-D-glucopyranoside	1.9 ^f^	Glucoside	Warner *et al*. [24]
1-Decyl-β-D-glucopyranoside	3.1 ^f^	Glucoside	Warner *et al*. [24]
1,2-Dihydroxypropyl octanoate	2.4 ^f^	Monoglyceride	Warner *et al*. [24]
1,2-Dihydroxypropyl decanoate	3.1 ^f^	Monoglyceride	Warner *et al*. [24]
1-Butyl-2-piperidinone	1.4 ^f^	Piperidinone	Warner *et al*. [24]
1-Hexyl-2-piperidinone	2.6 ^f^	Piperidinone	Warner *et al*. [24]
1-Octyl-2-piperidinone	3.4 ^f^	Piperidinone	Warner *et al*. [24]
1-Ethyl-2-pyrrolidone	−0.04 ^d^	Pyrrolidone	Yoneto *et al*. [22,45]
1-Butyl-2-pyrrolidones	1.02 ^f^	Pyrrolidone	Yoneto *et al*. [22,45]
1-Hexyl-2-pyrrolidinone	2.1 ^f^	Pyrrolidone	Yoneto *et al*. [22] He *et al*. [25], Ibrahim and Li [40]
1-Octyl-2-pyrrolidinone	3.0 ^f^	Pyrrolidone	Yoneto *et al*. [22] He *et al*. [25], Ibrahim and Li [40,56]
1-Decyl-2-pyrrolidinone	4.1 ^l^	Pyrrolidone	Warner *et al*. [39]
1-Dodecyl-2-pyrrolidinone	4.9 ^l^	Pyrrolidone	Warner *et al*. [39]
Thymol	2.5 ^m^	Terpene	Chantasart *et al*. [31]
Menthol	3.4 ^m^	Terpene	Chantasart *et al*. [31]
Menthone	2.9 ^m^	Terpene	Chantasart *et al*. [31] Ibrahim and Li [40]
Carvacrol	2.5 ^m^	Terpene	Chantasart *et al*. [31]
Cineole	3.1 ^m^	Terpene	Chantasart *et al*. [31]
1,2,3-Nonanetriol	1.6 ^f^	Triol	Warner *et al*. [24]

^a^ Values from the literature [57]; ^b^ Experimental values from the literature [27]; ^c^ Experimental values from the literature [58]; ^d^ Calculated values based on the chemical structure of the compound, obtained from EPI suite database; ^e^ Experimental values from the literature [26]; ^f^ Experimental values from the literature [24]; ^g^ Experimental values from the literature [23]; ^h^ Experimental values from the literature [59]; ^i^ Experimental values from the literature [30]; ^j^ Experimental values from the literature [60]; ^k^ Experimental values from the literature [61]; ^l^ Experimental values from the literature [39]; ^m^ Experimental values from the literature [31].

### 3.1. Enhancer Potency Based on Concentration in Aqueous Solutions and n-Octanol-PBS Partition Coefficients

**Figure 4:**
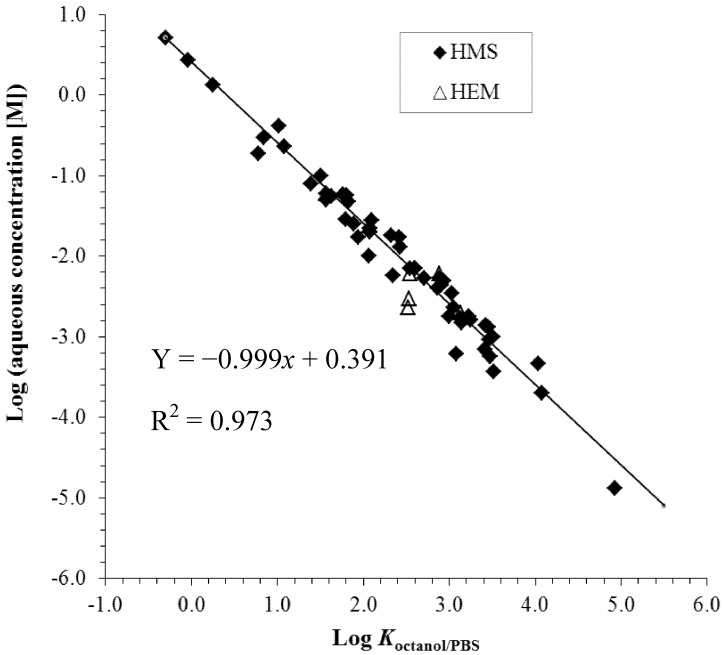
Relationship between the aqueous concentrations of the enhancers to induce 10-fold penetration enhancement and enhancer *K*_octanol/PBS_ (octanol/PBS partition coefficient). The data are taken from previous hairless mouse skin (HMS) and human epidermal membrane (HEM) studies [6,22,23,24,25,26,27,30,31]. PBS = phosphate buffered saline.

Studies using the symmetric and equilibrium approach to establish a quantitative structure enhancement relationship of penetration enhancers upon the skin lipoidal pathway have shown good correlation between the potencies of enhancers based on their concentration in aqueous solutions and enhancer n-octanol-PBS partition coefficients [23,24,25,26,27,31]. Figure 4 presents a plot of the aqueous concentrations of the enhancers to induce 10-fold permeation enhancement over the control under the symmetric conditions versus the octanol/PBS partition coefficients (*K*_octanol/PBS_) of the enhancers. As expected according to the discussion in the previous section, there was no significant difference between the HEM and HMS data. With a few moderate outliers, the data of all enhancers essentially fall on the same line with a slope close to −1. This shows a general relationship between enhancer potencies for transport enhancement across the stratum corneum lipoidal pathway and enhancer lipophilicities (enhancer *K*_octanol/PBS_) based on the concentration of the enhancers in the diffusion cell chambers. It is hypothesized that this relationship is a result of the partition tendency of the enhancers from the aqueous phase in the diffusion chambers to the stratum corneum lipid lamellae. Importantly, these findings provide a general quantitative structure-enhancement relationship based on the enhancer aqueous concentration, implying that the *K*_octanol/PBS_ is an excellent predictor of enhancer potency under the experimental condition employed and for the skin penetration enhancers studied to date. These results are also consistent with the hypothesis that the chemical microenvironment of the enhancer site of action in the stratum corneum lipoidal pathway is similar to that of water-saturated n-octanol; the slope of close to −1 is expected to occur only when the polarity of solvent n-octanol closely resembles that of the stratum corneum domain in which the enhancers are involved to induce penetration enhancement. This will be further discussed in the section “Microenvironment of the site of action of skin penetration enhancers.” To summarize, the study of the relationship between enhancer physicochemical properties and enhancer potency suggests that the effectiveness of the penetration enhancers based on their aqueous concentration in the diffusion cell chambers is related to their lipophilicity and their ability to partition into the transport rate-limiting domain in the stratum corneum intercellular lipid lamellae. 

### 3.2. Enhancer Potency Based on Concentration in the Stratum Corneum Lipids

**Figure 5:**
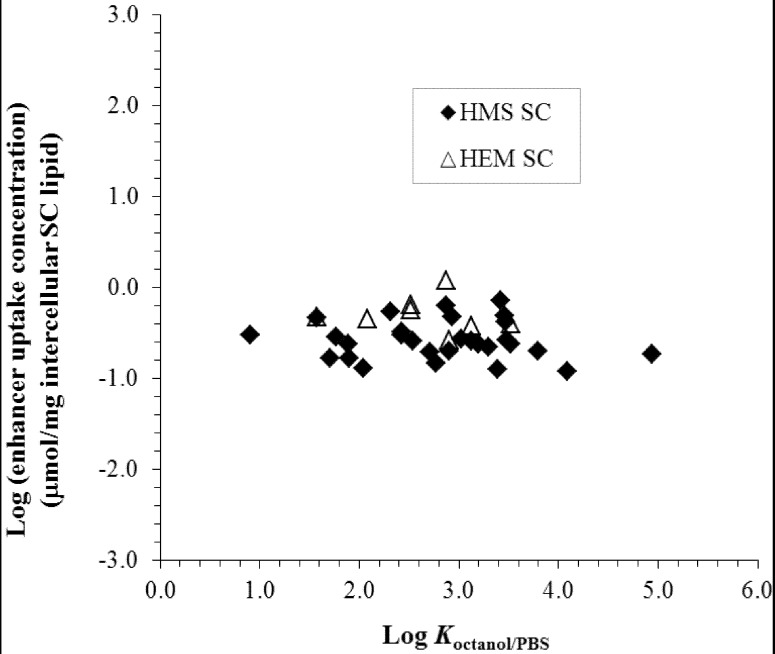
Relationship between enhancer uptake into hairless mouse skin stratum corneum (HMS SC) and human epidermal membrane stratum corneum (HEM SC) (μmol/mg intercellular SC lipid) under the aqueous enhancer concentration to induce 10-fold penetration enhancement and enhancer *K*_octanol/PBS_ (octanol/PBS partition coefficient). The data are taken from previous hairless mouse skin (HMS) and human epidermal membrane (HEM) studies [25,26,27,30,31]. PBS = phosphate buffered saline.

To establish a quantitative structure enhancement relationship based on enhancer concentration in the stratum corneum intercellular lipids, the concentration of enhancers in the stratum corneum intercellular lipids was investigated [25,26,27,30,31]. Figure 5 presents the concentration of the enhancers in the stratum corneum lipid lamellae, presumably at their site of action in the stratum corneum lipoidal pathway, under the condition when the enhancers induce 10-fold skin penetration enhancement. No significant difference was observed between the HEM and HMS data as discussed earlier. In addition, the concentration of the enhancers in the stratum corneum to induce 10-fold flux enhancement (or the amounts of enhancers required to induce the penetration enhancement) is relatively independent of enhancer *K*_octanol/PBS_. Despite some differences in the results among all the enhancers studied, the pattern in the figures suggests that the intrinsic potencies of the enhancers are within the same order of magnitude based on their concentration in the stratum corneum lipids, unlike the pattern shown in Figure 4. Together with the data in the preceding section, these findings suggest that (a) the intrinsic potencies of the enhancers at their site of action are relatively independent of their alkyl group chain length and the nature of their polar head groups; and (b) the lipophilicities of the enhancers mainly assist the translocation of the enhancers to their site of action through a free energy of transfer from the bulk aqueous phase to the action site. 

### 3.3. Relationship between Enhancer Maximum Enhancement Effect and Enhancer Concentration at the Site of Action

**Figure 6:**
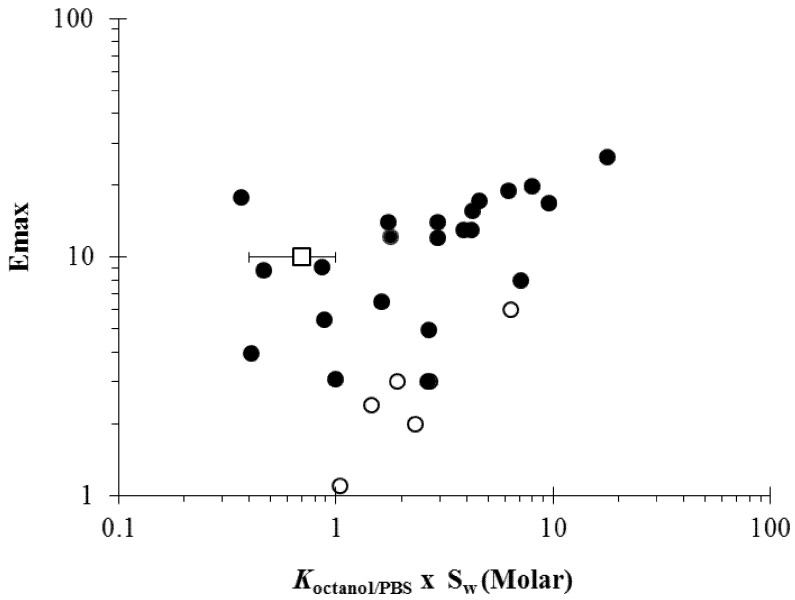
Relationship between Emax (the maximum enhancement effects) and the product of *K*_octanol/PBS_ (octanol/PBS partition coefficient) and Sw (aqueous solubility of enhancer) of the penetration enhancers (closed circles: enhancers, liquid at 37 °C; open circles: enhancers, solid at 37 °C). The average lipid concentration of enhancers to induce *E* = 10 in the symmetric studies, in which the enhancers were not at saturation, is also presented for comparison (open square). The data are taken from previous human epidermal membrane (HEM) studies [40,41].

As discussed in the section “Solvent systems in penetration enhancer studies”, a different experimental design is required to evaluate the potency of highly lipophilic penetration enhancers [40,41]. With this experimental design, the term “maximum penetration enhancement effect” or “Emax” was defined as the penetration enhancement effect (enhancement factor) induced by an enhancer upon the stratum corneum lipoidal pathway as the enhancer approaches the thermodynamic activity, equivalent to its pure state in equilibrium with the stratum corneum, in the absence of cosolvents or solubilizing agents. Experiments were performed by treatment of the skin with direct contact of the enhancer for equilibration. Thus, an enhancer with a higher thermodynamic energy state in its pure physical form would provide a higher concentration of the enhancer in the stratum corneum lipids. The structure enhancement relationship of lipophilic chemical enhancers was investigated under this condition. Figure 6 shows a relationship between Emax (*i.e.*, the maximum enhancement effects or enhancement factors) and the product of *K*_octanol/PBS_ and Sw (aqueous solubility of enhancer); the product *K*_octanol/PBS_ × Sw is the hypothetical enhancer solubility in n-octanol and is expected to correlate with the solubility of the enhancers in the stratum corneum lipid domain if the semipolar microenvironment of the enhancer site of action resembles the properties of n-octanol. For the enhancers in the liquid state at 37 °C and despite the data scatter due to the uncertainties of the *K*_octanol/PBS_ and Sw values obtained from the literature, the figure shows a trend of increasing Emax with an increase in *K*_octanol/PBS_ × Sw. This suggests that the effectiveness of the enhancers (or maximum enhancement effects, Emax) on penetration enhancement across the stratum corneum lipoidal pathway is related to the solubility of the enhancers in the stratum corneum lipid domain, consistent with the hypothesis that enhancer concentration in the lipid domain is a key factor to transdermal penetration enhancement. This also implies that the potencies of the enhancers compared at equal concentration in the stratum corneum lipids are not significantly different—a prerequisite for the observed correlation between enhancer solubility (or equilibrium concentration) in stratum corneum and Emax. For the enhancers in the solid state at 37 °C, the data demonstrate a correlation between Emax and *K*_octanol/PBS_ × Sw that is different from the liquid enhancers. The solid enhancers generally have lower Emax values than those of the liquid enhancers, suggesting that the solid enhancers have smaller maximum penetration enhancement effects and lower intrinsic potencies than those of the liquid enhancers. Also presented in the figure is the average concentration of enhancers in the stratum corneum lipids that induce 10-fold penetration enhancement under the conditions in the symmetric studies from Figure 5. This E = 10 enhancer concentration is within the data scatter, albeit in the higher range, of the general Emax versus *K*_octanol/PBS_ × Sw relationship in the figure. 

**Figure 7:**
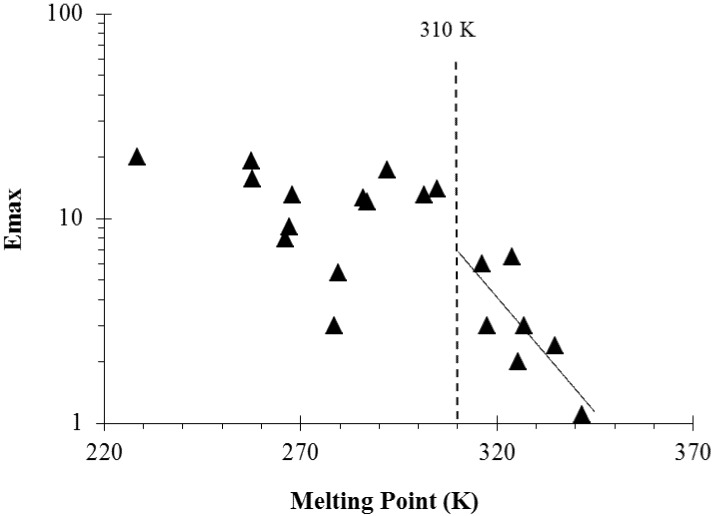
Maximum enhancement effects (Emax) versus the melting points of penetration enhancers. The dotted line separates the enhancers in liquid and solid states at 37 °C. The solid line is the linear regression line indicating a relationship between Emax and melting points of the solid fatty acid enhancers. The data are taken from Ibrahim *et al*. [40,41].

**Figure 8:**
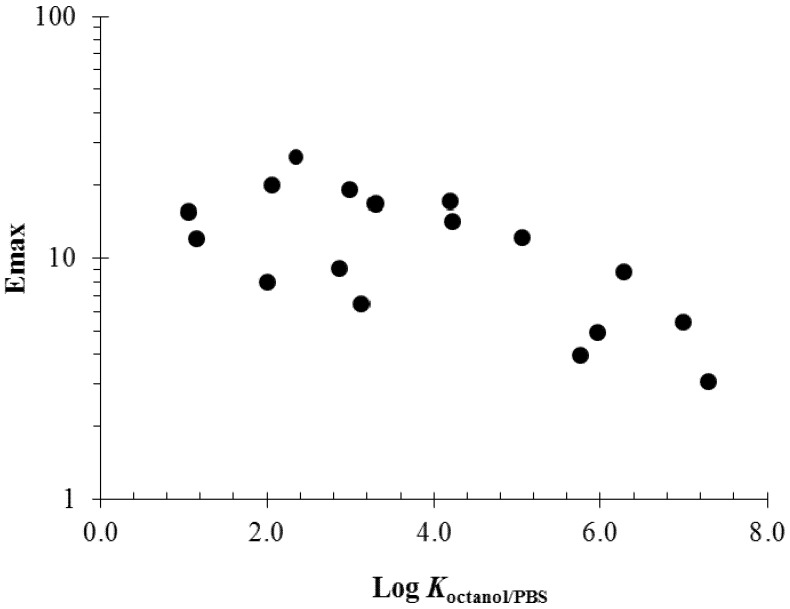
Relationship between maximum enhancement effects (Emax) and Log *K*_octanol/PBS_ of the enhancers. The data are taken from previous human epidermal membrane (HEM) studies [40].

Another observation in our enhancer studies is the relationship between the maximum enhancement effects (Emax) of the enhancers and their melting points [41] and *K*_octanol/PBS_ values [40]. The relationship between enhancer Emax and melting point is due to the fact that solute solubility is a function of solute melting point (*i.e.*, solid crystallinity). For example, enhancers of saturated alkyl chains or of longer alkyl chains have higher melting points and therefore provide less maximum enhancement effects. Figure 7 presents a plot of Emax against the melting points of the enhancers studied. For the solid enhancers at 37 °C (right side of the dotted line in the plot), an exponential function relationship of Emax versus enhancer melting point was found (see best fit line). This is consistent with the hypothesis that the enhancers of higher solubility, *i.e.*, higher equilibrium concentration in the stratum corneum induces larger maximum penetration enhancement. In addition, this suggests stronger penetration enhancement would be induced by the liquid enhancers compared to the solid enhancers. Figure 8 shows the relationship between Emax and *K*_octanol/PBS_ of the enhancers. Charged enhancers such as fatty acids in PBS are excluded in this analysis due to the effect of ion dissociation upon octanol/PBS distribution. According to the data in the figure, enhancer Emax decreases with increasing enhancer *K*_octanol/PBS_. Combining this observation with that in Figure 4, it can be concluded that enhancers with high *K*_octanol/PBS_ values are generally less potent skin penetration enhancers in terms of their maximum penetration enhancement effects but are more potent when compared at equal concentration in the diffusion cell chambers. 

### 3.4. Microenvironment of the Site of Action of Skin Penetration Enhancers

An observation in the enhancer studies of the symmetric and equilibrium approach is that the organic solvent n-octanol has similar polarity to that of the microenvironment of the enhancer site of action. This is supported by Figure 3, which demonstrates a relationship between the partition coefficient of enhancer partitioning between the aqueous phase and the stratum corneum intercellular lipid domain (*K*_SC__lipid/PBS_) and *K*_octanol/PBS_ [25,26,27,30,31]. The correlation in the figure is consistent with the hypothesis that the microenvironment of the enhancer site of action at the transport rate-limiting domain is well-mimicked by liquid n-octanol for the chemical enhancers studied. Within the data scatter, the data falling closely on the observed trend in the figure suggest that the microenvironment of the enhancer site of action in the stratum corneum is essentially the same for these enhancers, which have *K*_octanol/PBS_ values over a four-order of magnitude range. The correlation between enhancer maximum enhancement effects and the hypothetical enhancer solubilities in skin lipids shown in Figure 6 also supports the hypothesis that the microenvironment of the site of enhancer action has similar polarity to that of n-octanol. Although the generalization of the microenvironment of the enhancer site of action is over-simplistic because the stratum corneum lipid lamellae is not expected to behave as a conventional liquid homogeneous phase, the findings with the enhancers under the experimental conditions studied to date support the argument that a relationship likely exists between the n-octanol phase, the stratum corneum transport rate-limiting domain, and the microenvironment of the enhancer site of action in stratum corneum. 

## 4. Possible Mechanism of Action of Skin Penetration Enhancers

The findings discussed in the present review so far are on the effects of the skin penetration enhancers upon flux enhancement across the stratum corneum lipoidal pathway and the quantitative structure enhancement relationship of the enhancers on the macroscopic level. It was also concluded from these findings that the site of enhancer action is likely to be the stratum corneum lipid domain with polarity similar to n-octanol. To further examine the mechanism of action of skin penetration enhancers, the effects of the enhancers upon the microscopic behavior of stratum corneum lipids were investigated. 

Stratum corneum, the superficial layer of the skin, consists of corneocytes embedded in the intercellular lipid matrix, which are responsible for preventing the ingress of foreign molecules and the egress of endogenous substances. It is widely believed that the intercellular route provides the principal pathway for the penetration of most compounds [42,43]. A mechanism of skin penetration enhancers to promote transdermal drug penetration is to modify the properties of the stratum corneum intercellular lipids. Therefore, it is necessary to investigate the microstructure of the intercellular lipids in the stratum corneum. Recently, the lipid organization and microstructure of stratum corneum were studied using a number of techniques including fluorescence spectroscopy [44,45], differential scanning calorimetry (DSC) [46], and Fourier transform infrared (FTIR) [47,48]. These techniques were also used to determine the mechanisms of action of skin penetration enhancers. This section summarizes the findings in our enhancer structure activity relationship studies using these techniques. 

Fluorescence spectroscopy is commonly used to provide information on the microenvironment (such as lipid fluidity) of stratum corneum lipid lamella. Previous fluorescence probe studies on the influence of the short chain n-alkanols (*i.e.*, methanol, ethanol, 1-propanol, and 1-butanol) and of the 1-alkyl-2-pyrrolidones (*i.e.*, 1-ethyl-2-pyrrolidone, 1-butyl-2-pyrrolidone, 1-hexyl-2-pyrrolidone, and 1-octyl-2-pyrrolidone) on the lipid fluidity parameters (as assessed by the 9-anthroyloxy fatty acid fluorescent probes) under the conditions that induce 10-fold penetration enhancement of the lipoidal pathway over the control using stratum corneum lipid liposomes (SCLLs), have demonstrated a strong correlation between fluidity increases and penetration enhancement induced by the skin penetration enhancers [44,45]. The results of fluorescence anisotropy studies [44,45] and skin penetration enhancer studies [6,22] suggest that a probable mode of action of short chain n-alkanols and 1-alkyl-2-pyrrolidones in the enhancement of the lipoidal pathway for skin penetration involves the intermediate depth region (C2 to C9) of the lipids near the polar head plane, where the lipid alkyl chains are highly ordered and densely packed, rather than the deep hydrophobic interior of the lipid lamella. The intercalation/partition of the short chain n-alkanols and 1-alkyl-2-pyrrolidones into the intercellular lipids of stratum corneum is believed to be a critical part of the mechanism of action of penetration enhancement.

DSC has traditionally been used to provide information on the lipid conformation at the molecular level. In DSC studies of the stratum corneum intercellular lipids, stratum corneum phase transitions normally occur between 30 and 120 °C for both human and mouse stratum corneum [46]. In previous studies [49,50], typical *in vitro* analyses of isolated human stratum corneum have demonstrated 3 to 4 endothermic temperature transitions. The minor endothermic peak observed at ~35 °C has been attributed to the lower melting point lipids (*i.e.*, sebaceous lipids) [46] or lipid lamellae phase transition from a crystalline phase to a gel like phase [51]. The two major transitions ranging from 65 to 85 °C are likely to be associated with the lipids further changing from a gel phase to a more liquid phase [52]. At higher temperature ~95–105 °C, the endothermic peak is related to the denaturation of keratinocyte keratins [46,52]. A recent study performed by our group using DSC and human skin under the condition of the maximum enhancement approach investigated the phase transition behavior of enhancer-treated stratum corneum from 20–110 °C. The results showed a decrease in the phase transition temperature (between 70–85 °C) with an increase in Emax [40], suggesting that the increase in the gloss fluidity of the stratum corneum lipids is a mechanism of action of the skin penetration enhancers. This finding is consistent with the general view that skin penetration enhancement induced by chemicals is a result of the alteration of lipid organization and an increase in lipid lamellae disorder in the stratum corneum. 

FTIR spectroscopy has been considered a useful tool for determining the molecular vibrations of compounds in the stratum corneum [53,54,55]. This technique has been used to evaluate the functional group interactions between skin penetration enhancers and the stratum corneum intercellular lipids. The primary lipid vibrational modes of interest related to the intercellular lipids in the stratum corneum are CH_2_ asymmetric (~2920 cm^−1^) and CH_2_ symmetric stretching (~2850 cm^−1^) [46]. Previous FTIR results on the effects of short chain alcohols (*i.e.*, isopropanol, 1-propanol, and 1-butanol) upon lipids in SCLLs have suggested that short chain alcohols as penetration enhancers induce chain disorder and increase the fluidity of liquid crystalline lipids [48]. A similar FTIR study has further suggested that the mechanism of action of the skin penetration enhancers is through enhancer intercalation into stratum corneum intercellular lipids and subsequent lipid lamellae fluidization related to enhancer lipid concentration in the stratum corneum [56]. These results are consistent with the enhancer mechanism of enhancer intercalation into stratum corneum intercellular lipids and subsequent lipid lamellae fluidization related to enhancer concentration in the stratum corneum lipids.

## 5. Conclusion

Recent studies from our group based on previous experimental approaches (e.g. parallel pathway model and aqueous solution systems under the symmetric condition) continuing the effort of the previous skin penetration enhancer studies, have provided insights into the quantitative structure enhancement relationships of skin penetration enhancers and improved our understanding of the effectiveness of the enhancers for transport across the stratum corneum lipoidal pathway. Particularly, our recent studies examined the validity of hairless mouse skin as a model for human skin in penetration enhancer studies and showed a correlation between the hairless mouse skin and human skin results. The relationship between the effects of the enhancers under the symmetric and asymmetric conditions was also evaluated and the utility of the symmetric approach to study skin penetration enhancement was illustrated. Our studies of skin penetration enhancers continue to support the hypothesis that the potencies of the enhancers based on their concentration in the aqueous solutions in the diffusion cell chambers depend strongly on the lipophilicities (*i.e.*, octanol-water partition coefficients) of the enhancers. Furthermore, the intrinsic potencies of these enhancers based on their concentrations in the stratum corneum lipids are essentially the same (all within the same order of magnitude) with the enhancers of higher stratum corneum lipid solubilities capable of providing larger maximum transport enhancement effects. Our recent data are also consistent with the previous finding that the microenvironment of the enhancer site of action can be well mimicked by n-octanol. Finally, the mechanism of action of the enhancers is related to the fluidization of the intercellular lipids in the stratum corneum, supporting the hypothesis that the enhancer site of action is in the semi-polar region of the stratum corneum intercellular lipid domain for drug penetration across the stratum corneum lipoidal pathway.
