# Correction: Chantasart et al. “Structure Enhancement Relationship of Chemical Penetration Enhancers in Drug Transport across the Stratum Corneum” *Pharmaceutics* 2012, *4*, 71–92

**DOI:** 10.3390/pharmaceutics12010004

**Published:** 2019-12-18

**Authors:** Doungdaw Chantasart, S. Kevin Li

**Affiliations:** 1 Department of Pharmacy, Faculty of Pharmacy, Mahidol University, Bangkok 10400, Thailand; doungdaw.cha@mahidol.ac.th; 2 Division of Pharmaceutical Sciences, College of Pharmacy, University of Cincinnati, Cincinnati, OH 45267, USA

The authors wish to make the following corrections to their paper [1].

The values Log *K*_octanol/water_ of thymol and carvacrol in Table 1 (column 2, line 6 and line 3 from the bottom, respectively) are incorrect. With the correct values, the equation in Figure 4 (page 81) is also incorrect. The details of the corrections are described below.

1. Page 80: The correct values for the Log *K*_octanol/water_ of thymol and carvacrol [2] in Table 1 are 3.30 and 3.49, respectively.
2. Page 80: A new footnote (footnote n) has been added in Table 1.
3. Page 81: The correct equation and R^2^ in Figure 4 are y = −0.982*x* + 0.371 and R^2^ = 0.965, respectively.
4. Page 92: A new reference (Reference 62) has been added [2].

These changes do not affect the conclusion of the paper. The authors would like to apologize for any inconvenience this might have caused. 
